# Supplementary material for: Single-molecule imaging of transcription dynamics, RNA localization and fate in human T cells
Source: EMBO J. 2025 Oct 14;44(22):6732–49. doi: 10.1038/s44318-025-00592-0 (PMC12624010; doi:10.1038/s44318-025-00592-0)
Supplement: Supplementary file 1 — Appendix [file 44318_2025_592_MOESM1_ESM.pdf]

Appendix for

Single molecule imaging of transcription dynamics, RNA localization and fate in human T cells

Maria Valeria Lattanzio *et al.*

*Corresponding author. evelina.tutucci@vu.nl | m.wolkers@sanquin.nl

This PDF file includes:

Appendix Figure S1 (page 2)
Appendix Figure S2 (page 4)
Appendix Figure S3 (page 5)
Appendix Figure S4 (page 7)
Appendix Figure S5 (page 9)
Appendix Figure S6 (page 11)
Appendix Tables S1 (page 12)
Appendix Tables S2 (page 14)
Appendix Tables S3 (page 15)
Appendix Methods (page 16)

Appendix Fig. S1.

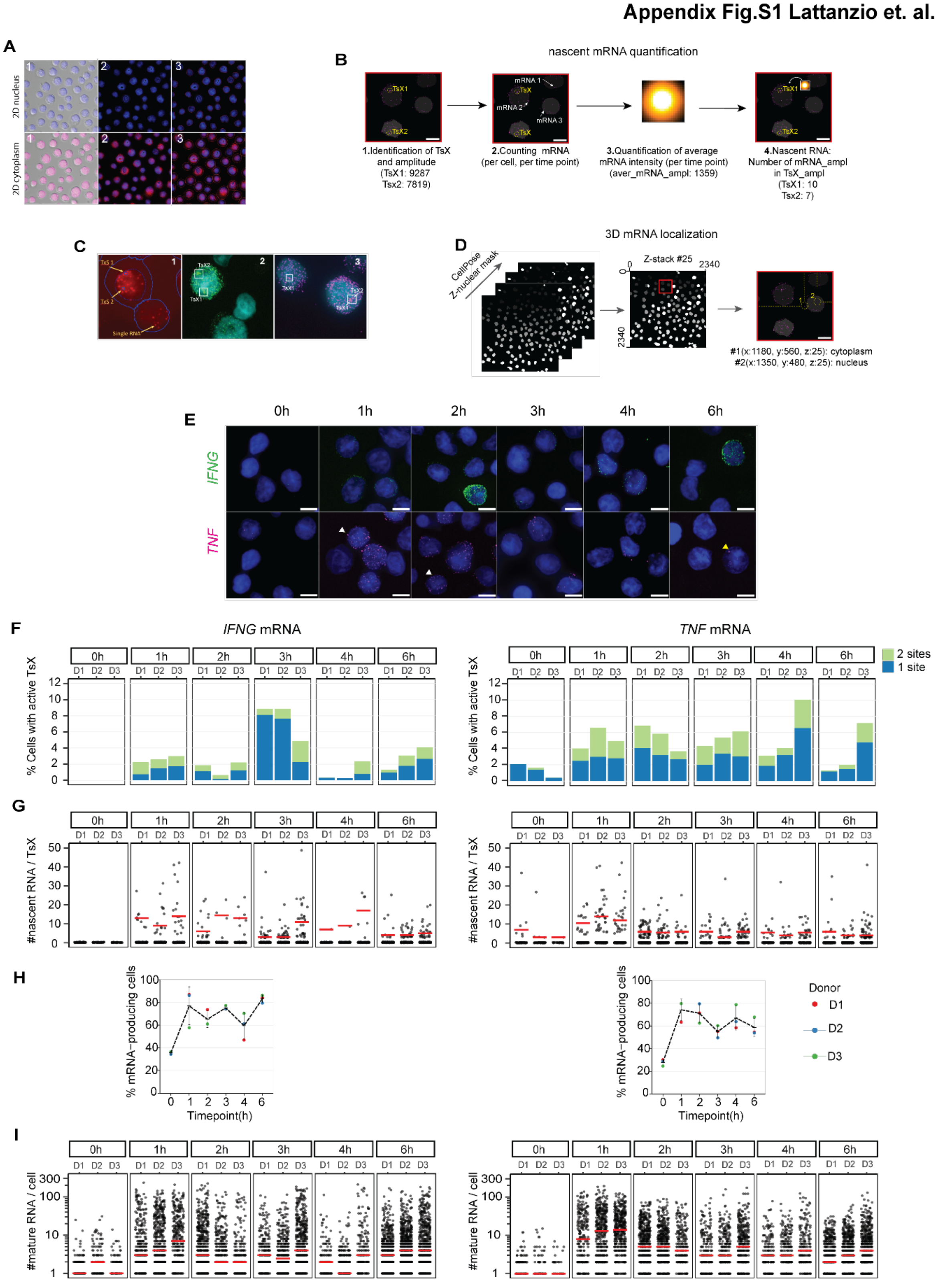

Appendix Fig. S1. *IFNG* and *TNF* mRNA quantification with T-cell smFISH

A. Schematic representation of 2D workflow to define nucleus (top panel) and cytoplasm (bottom panel) with fq-segmentation (FISH-quant). (1) Merged MAX projection of DIC with DAPI (nucleus, blue) or autofluorescence in the Quasar-670 channel (cell mask, magenta). (2) MAX projection of DAPI (top) and Quasar-670 (bottom) as input for fq-segmentation. (3) Nuclear (top) and cellular (bottom) outline depicted from fq-segmentation, indicated with red line. **B.** Scheme of nascent RNA quantification using FISH-quant. (1) TsXs are identified as high intensity fluorescent clusters overlapping with nuclear staining (DAPI). Their XYZ position and intensity (amplitude) are measured. (2) Cytoplasmic mRNAs are identified, their XYZ position and intensity is measured. (3) Quantification of average mRNA intensity and 3D size (amplitude and sigma XY, sigma Z) based on all mRNA measured at a given time point. (4) Estimation of the number of nascent RNA per transcription sites based on the amplitude of TsX and average mRNA. **C.** Maximal projection of $CD8^+$ T cells activated for 1h with α-CD3/α-CD28. Example of TsX identification and single mRNA using FISH-Quant Matlab (**1**) (blue line: 2D cellular outline, dotted blue line: 2D nuclear outline). Example of *IFNG* mRNA (green, **2**) and *TNF* mRNA (magenta,**3**) smFISH merged on DAPI staining (cyan). Boxes indicate the number of Transcription site (TsX1 or TsX 2). **D.** T-cell smFISH analysis pipeline. Z-stack nuclear masks (indicated in shades of gray) obtained with CellPose nuclear mask function. Pixel coordinates are extracted (range 0, 2340 pixels) per Z-nuclear mask. Mask #25 as an example. T-cell smFISH analysis extracts coordinate information to define localization of mRNA molecules inside or outside the nuclear/cytoplasmic mask, as exemplified for mRNA molecules in yellow dotted circles: mRNA#1: cytoplasmic, mRNA#2: nuclear. Scale bar: 5µm. **E.** Maximal projection of DAPI (blue), *IFNG* mRNA (top panel), and *TNF* mRNA (bottom panel) in Teff cells re-activated with α-CD3/α-CD28. White arrows: single mRNA molecules. Yellow arrows: TsX. Scale bar: 5 µm. **F.** Percentage of Teff cells with 1 (blue) or 2 (green) active transcription sites (TsX) for *IFNG* (left) and *TNF* (right) after α-CD3/α-CD28 stimulation. N=3 donors (D). **G.** Number of nascent *IFNG* (left) and *TNF* (right) RNA per active TsX. Each dot represents one TsX. n=3 donors. Red bar: median. **H.** Percentage of Teff cells expressing mature mRNA. n=3 donors, line: mean. **I.** Number of mature *IFNG* (left panel) and *TNF* (right panel) mRNA of Teff expressing ≥ 1 mature mRNA. Each dot represents one cell. n=3 donors. Red bar: median.

Appendix Fig. S2.

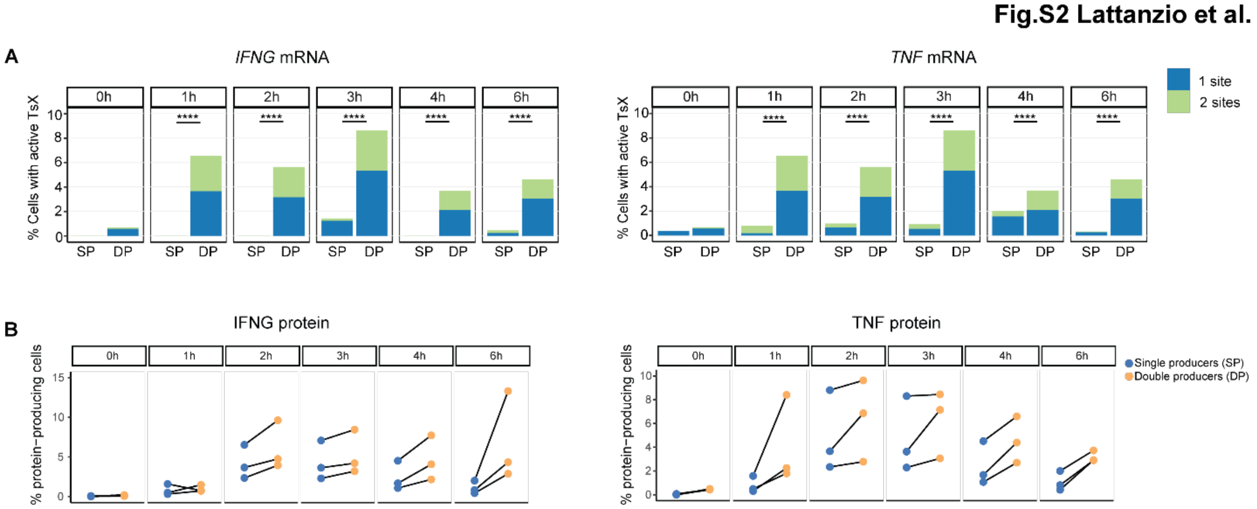

Appendix Fig. S2. ***IFNG* and *TNF* transcription in single and double positive expressors**
A. Percentage of Teff cells with one (SP, blue) or two (DP, green) active transcription sites for *IFNG* (left) and *TNF* (right) upon α-CD3/α-CD28 stimulation. Data depict median of 3 pooled donors. *p≤0.05, **p≤0.01, ***p≤0.001, ****p≤0.0001 ns: non-significant. Kruskal-Wallis non-parametric test, and post-hoc Tukey HSD test. For exact p-values, see Dataset EV1. **B.** IFN-γ (left) and TNF (right) protein expression of SP (blue) and DP (orange) protein-producing Teff cells. Bref A was added for a maximum of 2h of activation. Each dot indicates 1 donor.

Appendix Fig. S3.

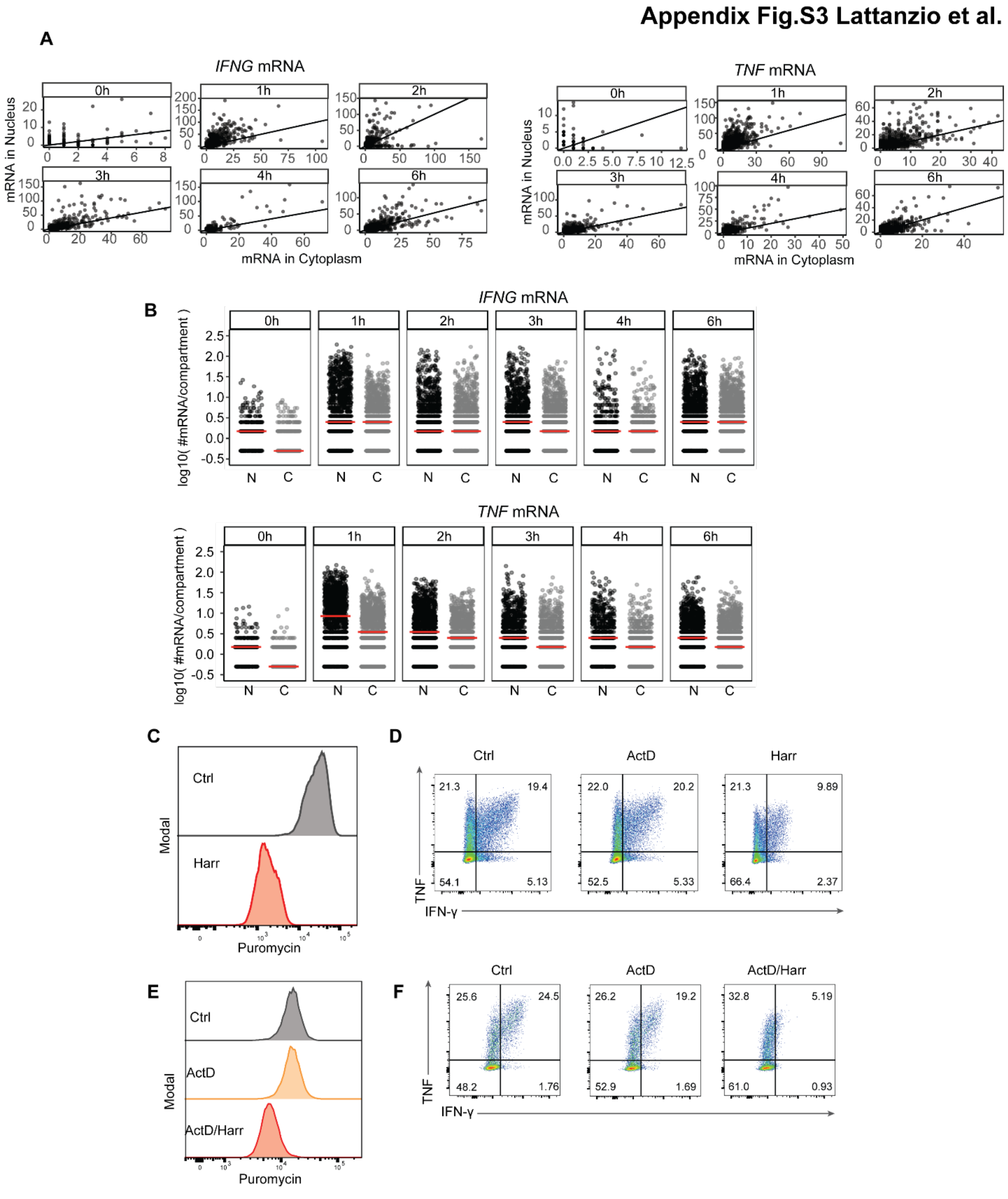

Appendix Fig. S3. IFNG and TNF expression depends on translation control

A. Scatter plot of cytoplasmic mRNA (x-axis) versus nuclear mRNA (y-axis) per cell of Teff cells expressing ≥1 mature mRNA. n ≥ 500 cells/time point, pooled from 3 donors. Black line: correlation line. **B.** Number of nuclear (N, grey) and cytoplasmic (C, black) cytokine mRNA in Teff cells expressing ≥ 1 mature RNA. Data representation in log10 scale (y-axis), with a coefficient of 0.5 added. Red bar: median **C.** Puromycin expression in Teff cells activated for 2h with α-CD3/α-CD28 that were treated for the last hour with Harringtonine (HARR) or left untreated (Ctrl). Puromycin was added for the last 10 min of T cell activation **D.** IFN-γ and TNF protein expression in Teff cells activated for 2h with α-CD3/α-CD28 that were treated for the 2nd hour with Actinomycin D (ActD), HARR, or left untreated. **E.** Puromycin expression in Teff cells activated for 2h with α-CD3/α-CD28 that were treated in the 2nd hour with indicated drug or left untreated (Ctrl). Depicted data representative for 3 donors. **F.** IFN-γ and TNF protein expression of Teff cells activated for 2h and treated in the 2nd hour with indicated drug. Data representative of 3 donors.

Appendix Fig. S4.

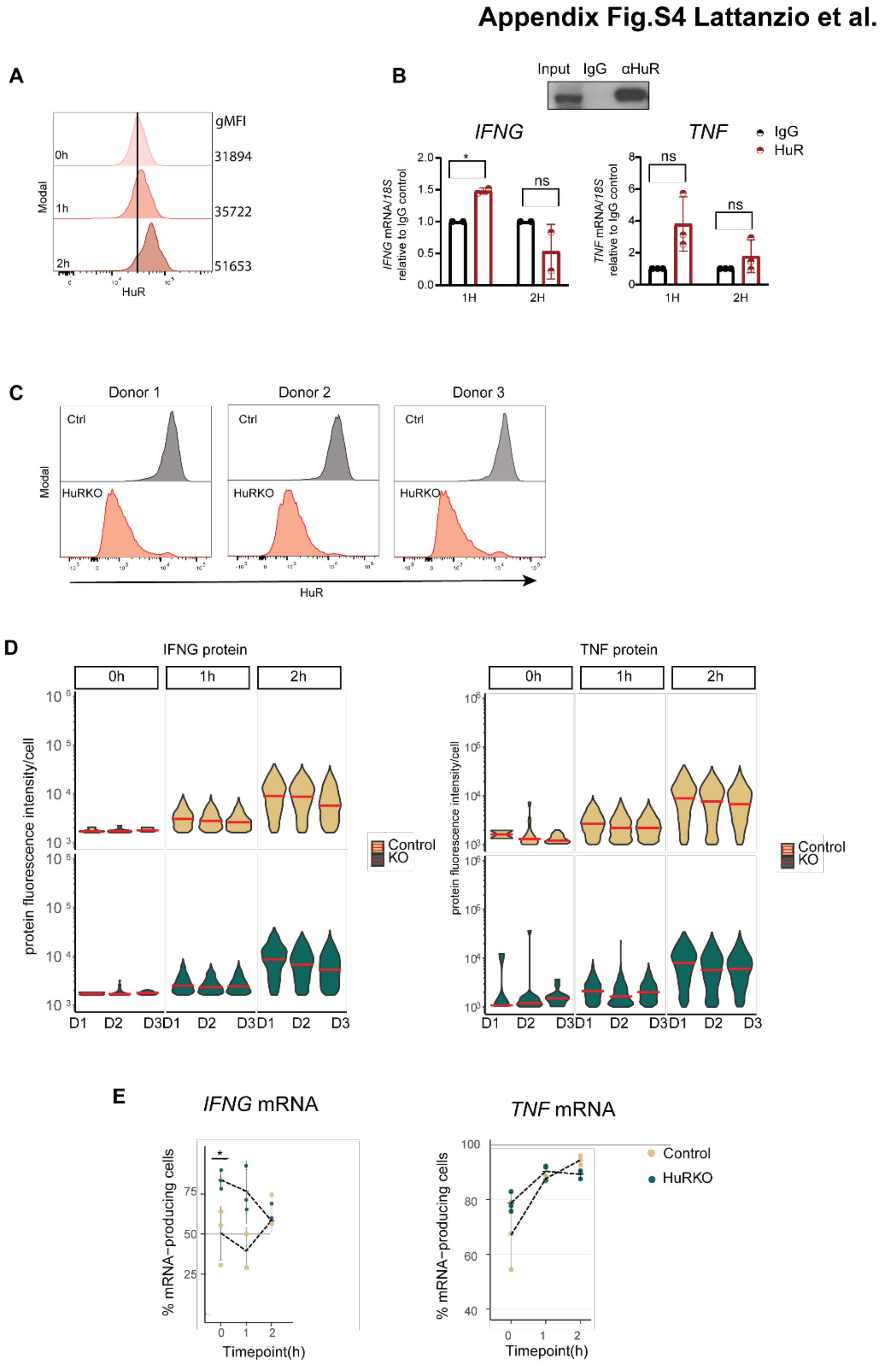

Appendix Fig. S4. Expression and interaction kinetics of HuR with *IFNG* and *TNF* mRNA

A. HuR protein expression in Teff cells that were resting (0h), or that were activated with α-CD3/α-CD28 for indicated time points. Geometric mean fluorescence intensity (gMFI) of HuR protein expression indicated on the right. Data representative of 3 donors. **B.** Native RNA

immunoprecipitation (RIP) with α-HuR or immunoglobulin G (IgG) isotype control from Teff cells activated for 1h and 2h with PMA-ionomycin. (Top) Immunoblot of HuR expresssion upon RIP. (Bottom) qRT-PCR of endogenous *IFNG* or *TNF* mRNA from RIP with HuR or IgG control antibodies. Data compiled of two (*IFNG*) or three (*TNF*) donors from independently performed experiments. Mean ± SD. *p≤0.05, **p≤0.01, ***p≤0.001, ****p≤0.0001 ns: non-significant. Two-tailed paired t-test. For exact p-values, see Dataset EV1. **C.** HuR expression in HuR-KO and control-nucleofected (Ctrl) Teff cells by flow cytometry. n=3 donors. **D.** Violin plot of protein fluorescence intensity per cell per each donor (D1, D2, D3) at indicated time points. Red bar: median expression. **E.** Percentage of Teff cells expressing ≥1 mature *IFNG* (left) and *TNF* (right) mRNA in Control and HuR KO Teff cells. Each dot indicates 1 donor. Line: mean. (*p≤0.05, post-hoc Tukey HSD. For exact p-values, see Dataset EV1).

Appendix Fig. S5.

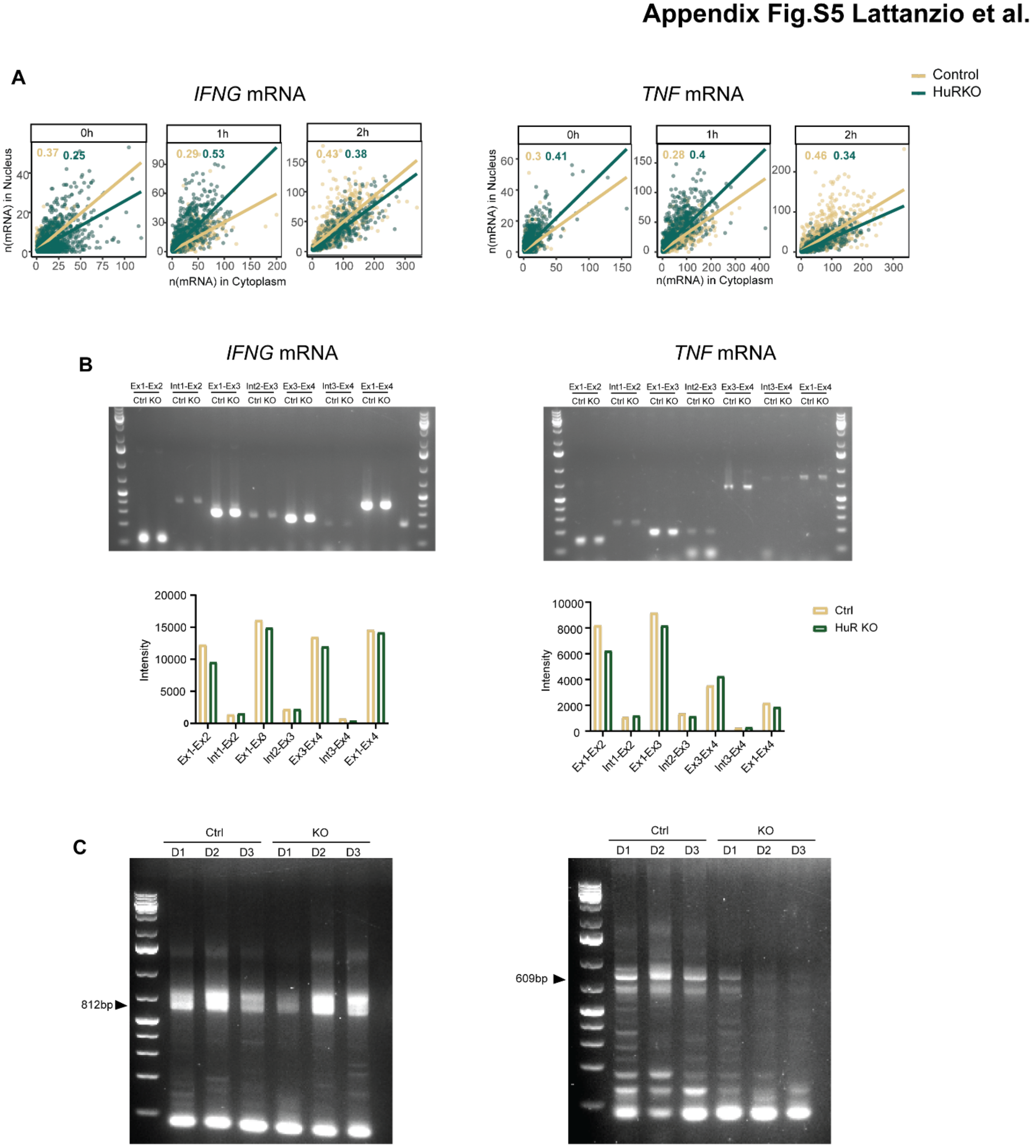

Appendix Fig. S5. Analysis of *IFNG* and *TNF* splicing and polyadenylation

A. Scatter plot of cytoplasmic (x-axis) versus nuclear mRNA (y-axis) per cell of Teff cells expressing ≥1 mature mRNA. n ≥ 500 cells/time point, pooled from 3 donors. **B.** Measurement of *IFNG* (left) and *TNF* (right) intron-exon junctions with RT-PCR of Control (Ctrl) and HuR KO

(KO) Teff cells that were stimulated for 1h with α-CD3/α-CD28. Top: RT-PCR gel. Bottom: quantification of band intensity. **C.** Measurement of poly(A) length of cytokine mRNA in control (Ctrl) or HuR-KO (KO) Teff cells activated for 1h with α-CD3/α-CD28 using RL-PAT assay. n=3 donors. Arrows indicate *IFNG* 3'UTR full-length: 812bp, and *TNF* 3'UTR full-length: 609bp.

Appendix Fig. S6

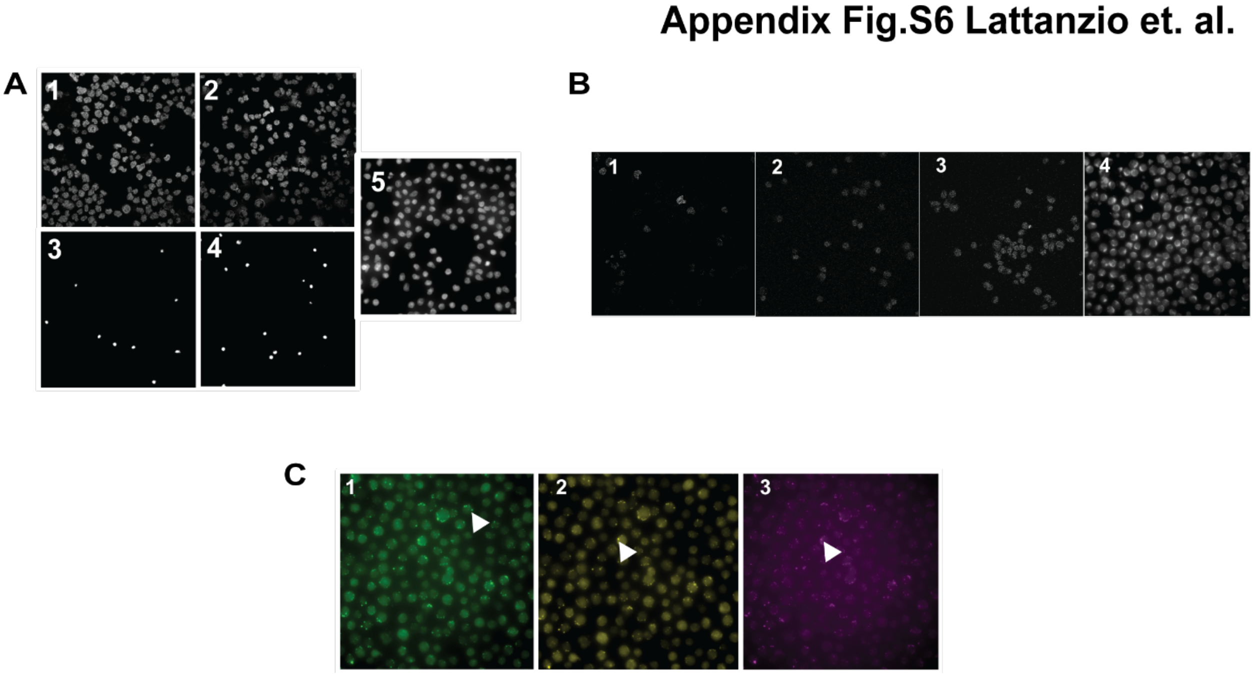

Appendix Fig. S6

A. Optimizing Coverslip coating. MAX projection of resting $CD3^+$ T cells on coverslips mechanically seeded with cytospin (2), or on cover slips coated with Corning® Cell-Tak™ Cell and Tissue Adhesive (1), Poly-L-lysine high MW (3), Poly-L-lysine MW (4), or Alcian Blue (5). T cells were stained with DAPI. **B.** MAX projection of resting CD3+ T cells on coverslips coated with Alcian Blue using different amount of cells: 250.000 cells (1), 500.000 cells (2), 750.000 cells (3) and $1x10^6$ T cells (4). The images are MAX projection of cellular autofluorescence in FITC channel. **C.** MAX projection of FITC channel (**1**), Cy3 (**2**) and Cy5 (**3**). White arrows indicate the autofluorescence signal in structures of dots.

Appendix Table S1.
***IFNG* and *TNF* probes for T cell smFISH**

human *IFNG* probes	human *TNF* probes
agctgatcttcagatgatca	ggtctgtagttgcttctctc
caggtccaaaggacttaact	cgtctgagggttgttttcag
gtagttcttgtatcaagctg	gggtcagtatgtgagaggaa
atttcatcgtttccgagaga	atcatgctttcagtgctcat
gcagagctgaaaagccaaga	aggctgaggaacaagcaccg
taacagccaagagaacccaa	tgccacgatcaggaaggaga
ttttacatatgggtcctggc	caaagtgcagcaggcagaag
atttcttaaggttttctgct	gagggctgattagagagagg
catctgaatgacctgcatta	cggggttcgagaagatgatc
aaaagagttccattatccgc	cttgagggtttgctacaaca
tatttttctgtcactctcct	accagctggttatctctcag
aggagacaatttggctctgc	ctgggagtagatgaggtaca
gctctggtcatctttaaagt	ctgatggtgtgggtgaggag
atggtctccacactcttttg	tctggtaggagacggcgatg
acttgacattcatgtcttcc	ctcttgatggcagagaggag
tcgtttctttttgttgctat	gatagatgggctcataccag
tagtcagcttttcgaagtca	attgatctcagcgctgagtc
attcaagtcagttaccgaat	caaagtcgagatagtcgggc
catgtattgctttgcgttgg	aaagtagacctgcccagact
tcagccatcacttggatgag	ctcctcacagggcaatgatc
tgttttagctgctggcgaca	ttgggaaggttggatgttcg
atctgactcctttttcgctt	ataaagggattggggcaggg
atgctcttcgacctcgaaac	ttgagggtgtctgaaggagg
aggcaggacaaccattactg	tctctttttgagccagaaga
agtgagacagtcacaggata	aagttctaagcttgggttcc
acatagccttgcctaattag	tcgaagtggtggtcttgttg
ccctgagataaagccttgta	cacacattcctgaatcccag

ttaggttggctgcctagttg	ttgaattcttagtggttgcc
aaacacacaacccatgggat	agggatcaaagctgtaggcc
gttcattgtatcatcaagtg	tggtctccagattccagatg
ctggatagtatcacttcact	cattctggccagaaccaaag
gcatattttcaaaccggcag	taggtgaggtcttctcaagt
aagttctgtctgacatgcca	aaggtccacttgtgtcaatt
atcagggtcacctgacacat	acatctggagagaggaaggc
tctcctgagatgctatgttt	cgtgtctcaaggaagtctgg
tttggaagcaccaggcatga	taaatagagggagctggctc
atgagttactttccatttgg	ccggtctcccaaataaatac
	caaggcagctcctacattgg
	agctccgttttcacggaaaa
	ctacatgggaacagcctatt
	caaaagaaggcacagaggcc
	ttggtcaccaaatcagcatt
	agaggctcagcaatgagtga
	gggcgattacagacacaact
	ctttatttctcgccactgaa

Appendix Table S2.

RT-PCR and qRT-PCR primers

**Reverse Transcription-PCR (RT-PCR)**	
**Primer name**	**Sequence**
F-IFNg_Ex1	CTGTTACTGCCAGGACCCAT
F-IFNg_Int1	GGGGCAGTATTTATAGTGGGG
R-IFNg_Ex2	GTTCCATTATCCGCTACATCTGA
F-IFNg_Int2	AAGCTGAATATTCCCATTTGGC
R-IFNg_Ex3	CAGCTTTTCGAAGTCATCTCGT
F-IFNg_Ex3	ATGCAGAGCCAAATTGTCTC
R-IFNg_Ex4	GCTTCCCTGTTTTAGCTGCT
F5-IFNg_Int3	TGACCATCATGACATTAGCAGA
R3-IFNg_Ex4	CTCTTCGACCTCGAAACAGC
F-TNFa_Ex1	TGCTTGTTCCTCAGCCTCTT
F2-TNFa_Int1	AAGGAGAGAGATGGGGGAGA
R-TNFa_Ex2	GGCCAGAGGGCTGATTAGAG
F-TNFa_Int2	GGTTTGGGGGTAGGGTTAGT
R-TNFa_Ex3	TGGGCTACAGGCTTGTCACT
F-TNFa_Ex3	GACAAGCCTGTAGCCCATGT
F-TNFa_Int3	GCACAGGCCTTAGTGGGATA
R2-TNFa_Ex4	AGGCCCCAGTTTGAATTCTT
**Real Time or Quantitative PCR (RT-qPCR)**	
IFNg RT2.0 FW	AGCTCTGCATCGTTTTGGGTT
IFNg RT2.0 RV	GTTCCATTATCCGCTACATCTGAA
TNFa Fw	GTTCCATTATCCGCTACATCTGAA
TNFa RV	TCAGCCTCTTCTCCTTCCTG
IL2 Fw	CAAGAATCCCAAACTCACCAG
IL2 RV	CGTTGATATTGCTGATTAAGTCC
18S_3F	GTGGAGCGATTTGTCTGGTT

18S_3R	AACGCCACTTGTCCCTCTAA

Appendix Table S3. RL-PAT primers

**RNA ligation-mediated poly(A) test (RL-PAT)**	
PAT-anchor	5’-rApp GGT CAC CTT GAT CTG AAG ddC-3’
PAT-R1	5’-GCT TCA GAT CAA GGT GAC CTT TTT-3’
PAT IFNy 3'UTR F	GGTTGTCCTGCCTGCAATATTTG
PAT TNF 3'UTR F	GGAGGACGAACATCCAACCTTC

Appendix Methods

Coverslip coating selection

For efficient T cell attachment, we compared different coating methods (**Appendix Fig. S6A)**: $CD8^+$ T cells were coated on round 16-mm coverslips (Fisherbrand Borosilicate Glass Circle Coverslip) using (**2**) Cytospin (3min x1800rpm), or on coverslips coated with (**2**) Poly-L-lysine high molecular weight (0.1 % (w/v) in H_2O, Sigma), **(3)** Poly-L-lysine low molecular weight (0.1 % (w/v) in H_2O, Sigma) (**4**), Corning® Cell-Tak(TM) Cell (**1**) and (**5**) Tissue Adhesive and Alcian Blue (Alcian blue in 3% acetic acid, Sigma). Corning® Cell-Tak™ (**1**) and mechanical seeding using cytospin (**2**) resulted in high-density seeding, but perturbed the shape of T cells. Coating with Poly-L-lysine (high MW (**3**) and low MW (**4**)) resulted in poor T cell attachment. With high T cell attachment and maintaining T cell shape, Alcian Blue (**5**) was most optimal for mounting T cells.

T cell amount for coating

To obtain sufficient $CD8^+$ T cells for smFISH measurements, we tested different numbers of $CD8^+$ T cells mounted onto Alcian Blue coated coverslips (**Appendix Fig. S6 B**). This titration essay showed that $1x10^6$ T cells was most optimal.

Fluorophore and filter setting selection

To obtain a high-quality smFISH signal, low background signal from cells is key. We found that autofluorescence signal of $CD3^+$ T cells presented with a dot shape (**Appendix Fig. S6 C;** see white arrows**)** in the FITC channel (**1**), Cy3 channel (**2**) and Cy5 channel (**3**). While these autofluorescence dots do not interfere with standard (protein) immunofluorescence, they can be mislabelled as single-molecule RNA. We therefore selected Cal-Fluo 610 (Cy3.5 channel) and Quasar-670 (Cy5 channel) as fluorophores conjugated to *IFNG* and *TNF* smFISH probes, respectively, and we used the AHF-LED-FISH-R Filter for Cy3.5 and the F36-523 Cy5 HCBrightLine Filter for Cy5 to reduce the background signal to increase the signal-to-noise ratio.
