# Supplementary material for: Single-molecule imaging of transcription dynamics, RNA localization and fate in human T cells
Source: EMBO J. 2025 Oct 14;44(22):6732–49. doi: 10.1038/s44318-025-00592-0 (PMC12624010; doi:10.1038/s44318-025-00592-0)
Supplement: Supplementary file 4 — Movie EV1 [file 44318_2025_592_MOESM4_ESM.zip › Movie_EV1/Movie_EV1.docx]

**Movie EV1**

3D model of acquisition field of view generated from T-cell smFISH with 61 Z-stacks. The video shows multiple Teff cells on coverslips. Blue: nucleus (DAPI), green: *IFNG* mRNA (CALFluorRed 610), magenta: *TNF* mRNA (Quasar-670). In the left corner, scale bar: 5μm, adjusting according to movie magnification.
