# Supplementary material for: Single-molecule imaging of transcription dynamics, RNA localization and fate in human T cells
Source: EMBO J. 2025 Oct 14;44(22):6732–49. doi: 10.1038/s44318-025-00592-0 (PMC12624010; doi:10.1038/s44318-025-00592-0)
Supplement: Supplementary file 5 — Movie EV2 [file 44318_2025_592_MOESM5_ESM.zip › Movie_EV2/Movie_EV2.docx]

**Movie EV2**

3D model of T-cell smFISH in three Teff cells. The video shows subsequentially staining for blue: nucleus (DAPI), green: *IFNG* mRNA (CALFluorRed 610), magenta: *TNF* mRNA (Quasar-670). Last image: merged. Scale bar: 5μm.
