# Supplementary figures and images for: Single-molecule imaging of transcription dynamics, RNA localization and fate in human T cells
Source: EMBO J. 2025 Oct 14;44(22):6732–49. doi: 10.1038/s44318-025-00592-0 (PMC12624010; doi:10.1038/s44318-025-00592-0)

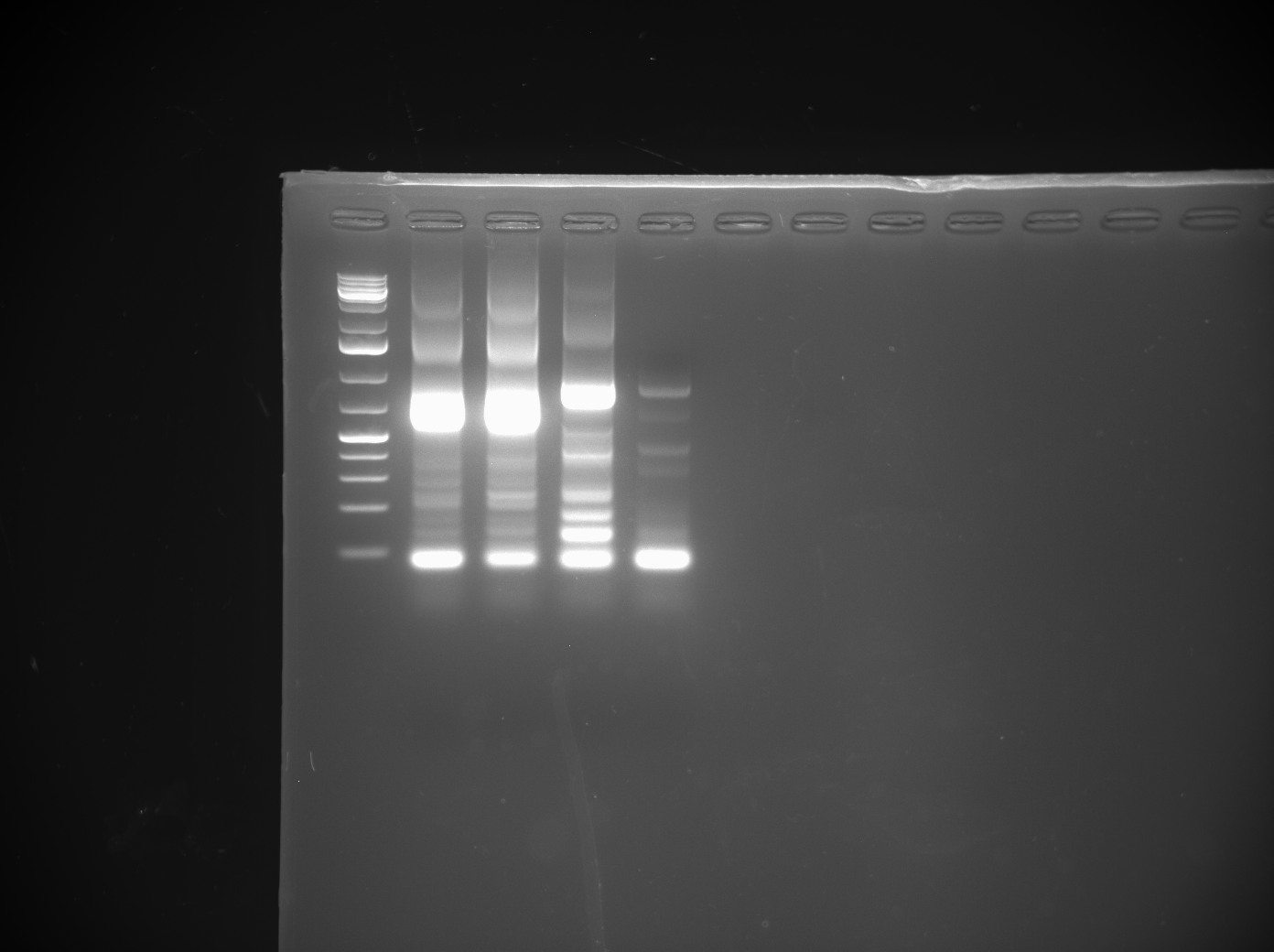

Supplement: Supplementary file 10 — Source data Fig. 5 [file 44318_2025_592_MOESM10_ESM.zip › Figure_5/5D/PAT-assay_TNF.tif]
